# Book Review: Societies Under Threat: A Pluri-Disciplinary Approach

**DOI:** 10.3389/fpsyg.2021.642260

**Published:** 2021-07-28

**Authors:** Petra Pelletier

**Affiliations:** ^1^University of Paris, Laboratory of Social Psychology (EA 4471), Paris, France; ^2^University of Limoges, Research Center for Semiotics (EA 3648), Limoges, France

**Keywords:** society, threat, pluri-disciplinarity, processes, coping, definition, concept

The unpredictable threats that societies are facing nowadays are related to various societal issues, such as terrorism, natural and man-made disasters, global health epidemics, financial breakdowns, refugee crisis, climate change, and so forth. Without doubt, the first edition of *Societies Under Threat: A Pluri-Disciplinary Approach* published in 2020 is an unprecedent masterpiece that unifies scholars from various disciplines, namely sociology, anthropology, psychology, philosophy, environmental sciences, and economics. In the collection of 15 chapters, the stimulating pluri-disciplinary approach attempts to seize the actual societal threats in their complexity. The volume also focuses on how these threats are constructed and how laymen and public instances deal with these contemporary threats.

The first edition of *Societies Under Threat: A Pluri-Disciplinary Approach* aims to renew the classic framework of contemporary societal issues by dealing with threats and other new phenomena that our societies are facing nowadays. The book that is composed of 15 chapters is divided into three specific parts that offer pluri-disciplinary perspectives on contemporary threats. Specifically, the first part of the book *Thinking Threats: Opening Views on Phenomena and Social Processes* that is composed of five chapters explores the meaning of the analytical category of threat and aims to confront and distinguish the concept of threat from similar concepts, such as risk, crisis, and catastrophe. The comparative analysis of the concept of threat and the concept of risk allows to rethink Beck's 1986/2008 theory of risk society and to develop new conceptual framework to better apprehend current societal phenomena. Moreover, this part of the volume highlights specific processes that contribute to the development of new taxonomy of contemporary societal phenomena. The second part of the volume *Building Threats: Cultures, Groups, and Identities* that is composed of five chapters aims to seize the cultural and identity processes that underpin the social construction of contemporary societal threats. More specifically, it concerns contemporary threats such as global terrorism, climate change, nationalism, refugee crisis, and threatening minorities that are ontologized as wild. The third and last part of the book *Confronting Threats in the Public Spere: Refusal, Change, Action* aims to seize the scope of consequences of contemporary threats. In particular, the collection of five chapters focuses on different reactions and strategies that allow laymen and public instances to deal with major contemporary phenomena, such as climate change, financial breakdowns, or global health epidemics.

As it is stated in the first part of the volume *Thinking Threats: Opening Views on Phenomena and Social Processes*, the concept of threat is generally associated with other fear-eliciting concepts, such as risk, crisis, catastrophe or disaster. Therefore, despite the fact that these notions have been studied since several decades in the field of humanities and social sciences, the current societal phenomena require irrevocably a pluri-disciplinary approach due to the increasing complexity of contemporary societies. Thus, the scientific concept of threat remains particularly vague and difficult to apprehend because this analytical category is constructed on various social, historical, religious, and societal factors. The threat approach framework exposed in the volume tends to integrate various approaches that relatively unambiguously deal with cataclysmic viewpoint of our actual and future societies. Accordingly, the pluri-disciplinary threat approach framework aims to begin an urgent reflection about the emergence of threats that our societies are facing nowadays. Indeed, the emerging societal threats seem to transcend the usual conceptual framework studied by risk researchers.

As a matter of fact, the concept of risk that has been studied since centuries in various disciplines offers a comprehensive framework of various risks that humanity is enduring since the beginning of humankind. In Modern history of science, the fate of the Gods has been progressively replaced by the science of probability that aims to quantify uncertainty, and therefore control the risk (Bernstein, 1996). Thus, the concept of risk refers to an objective calculation of risk probability combined with risk severity. In contrast to the conceptual framework of risk, the threat approach framework exposed in the volume posits that the category of phenomena named “risk” became insufficient. This insufficiency is primarily due to the increasing complexity of current societal issues, wherein the transcendental damages became unbounded in time and space. Furthermore, the threat approach framework posits that in the age of societal uncertainty, the production of meaning via social construction processes is the main defense against perceived threat and related feeling of fear. Nevertheless, the social psychological literature on threat also posits those various threats attempt to elicit specific anxiety-related processes and reactions (Jonas et al., 2014). Further, the omitted ontological anxiety, which is existential in nature, refers to the absolute threat of extinction of human being (Hendrix, 1967). As the contemporary threats exposed in the volume, the ontological anxiety that is unbounded in time and space tends to transcend our contemporary societies (Gustafsson and Krickel-Choi, 2020).

Therefore, the complexity and large-scale nature of emerging threats require proper defense perspectives. The primarily constructivist approach of the volume tends to omit the classic transactional model of stress and coping theory framework that is based on cognitive appraisals of the environment (Lazarus and Folkman, 1984). Specifically, “threat” refers to a stressful appraisal of an encounter that concerns anticipated harms or losses. Thus, contemporary threats that are directly challenging individual and collective resources require related coping strategies. Despite the fact that the volume does not focuses directly on these conceptual considerations, the chapters on collective denial that comes along with the Spanish flu epidemic (Chapter 15) and collective emotional synchronization that helps people to overcome collective traumas (Chapter 16) testify the relevance of such additional conceptual considerations.

In conclusion, the pluri-disciplinary approach of *Societies Under Threat* remains irrevocably audacious in many ways. The holistic integration of various definitional viewpoints, social construction processes and reactions that laymen and public instances deploy to deal with current threats remains particularly enriching. Although, a more systematic integration of knowledge that strives toward a transdisciplinary approach to contemporary threats appears as a necessary pathway for future societal research.

## Author Contributions

The author confirms being the sole contributor of this work and has approved it for publication.

## Conflict of Interest

The author declares that the research was conducted in the absence of any commercial or financial relationships that could be construed as a potential conflict of interest.

## Publisher's Note

All claims expressed in this article are solely those of the authors and do not necessarily represent those of their affiliated organizations, or those of the publisher, the editors and the reviewers. Any product that may be evaluated in this article, or claim that may be made by its manufacturer, is not guaranteed or endorsed by the publisher.

## References

[B1] Beck U. (1986/2008). La Société du Risque: Sur la Voie d'une Autre Modernité. [*Risk Society: Towards a New Modernity*]. Paris: Flammarion.

[B2] Bernstein P. L. (1996). Against the Gods: The Remarkable Story of Risk. New York, NY: John Wiley.

[B3] Gustafsson K. Krickel-Choi N. C. (2020). Returning to the roots of ontological security: insights from the existentialist anxiety literature. Eur. J. Int. Relat. 26, 875–895. 10.1177/1354066120927073

[B4] Hendrix H. (1967). The ontological character of anxiety. J. Relig. Health 6, 46–65. 10.1007/BF01533393 24424952

[B5] Jonas E. McGregor I. Klackl J. Agroskin D. Fritsche I. Holbrook C.. (2014). Threat and defense: from anxiety to approach, in Advances in Experimental Social Psychology, Vol. 49, eds Olson J. E. Zanna M. P. (San Diego, CA: Academic Press), 219–286.

[B6] Lazarus R. S. Folkman S. (1984). Stress, Appraisal, and Coping. New York, NY: Springer Publishing Company.

